# Inositol, neural tube closure and the prevention of neural tube defects

**DOI:** 10.1002/bdra.23533

**Published:** 2017-01-31

**Authors:** Nicholas D.E. Greene, Kit‐Yi Leung, Andrew J. Copp

**Affiliations:** ^1^ Newlife Birth Defects Research Centre and Developmental Biology & Cancer Programme, Institute of Child Health, University College LondonLondonUnited Kingdom

**Keywords:** neural tube defects, spina bifida, inositol, phosphoinositide, folic acid, clinical trial

## Abstract

Susceptibility to neural tube defects (NTDs), such as anencephaly and spina bifida is influenced by genetic and environmental factors including maternal nutrition. Maternal periconceptional supplementation with folic acid significantly reduces the risk of an NTD‐affected pregnancy, but does not prevent all NTDs, and “folic acid non‐responsive” NTDs continue to occur. Similarly, among mouse models of NTDs, some are responsive to folic acid but others are not.

Among nutritional factors, inositol deficiency causes cranial NTDs in mice while supplemental inositol prevents spinal and cranial NTDs in the *curly tail* (*Grhl3* hypomorph) mouse, rodent models of hyperglycemia or induced diabetes, and in a folate‐deficiency induced NTD model. NTDs also occur in mice lacking expression of certain inositol kinases. Inositol‐containing phospholipids (phosphoinositides) and soluble inositol phosphates mediate a range of functions, including intracellular signaling, interaction with cytoskeletal proteins, and regulation of membrane identity in trafficking and cell division.

*Myo*‐inositol has been trialed in humans for a range of conditions and appears safe for use in human pregnancy. In pilot studies in Italy and the United Kingdom, women took inositol together with folic acid preconceptionally, after one or more previous NTD‐affected pregnancies. In nonrandomized cohorts and a randomized double‐blind study in the United Kingdom, no recurrent NTDs were observed among 52 pregnancies reported to date.

Larger‐scale fully powered trials are needed to determine whether supplementation with inositol and folic acid would more effectively prevent NTDs than folic acid alone. Birth Defects Research 109:68–80, 2017. © 2016 The Authors Birth Defects Research Published by Wiley Periodicals, Inc.

## Introduction

### Maternal Nutrition during Pregnancy Influences Risk of NTDs

Failure in the process of neural tube closure during embryonic development results in severe birth defects termed neural tube defects, including anencephaly and spina bifida. Patterns of inheritance of NTDs indicate a major genetic contribution to risk of NTDs in the developing fetus, while it is also clear that non‐genetic, environmental, factors play a key role (Greene and Copp, 2014). These may include exogenous agents including anti‐epileptic drugs, such as valproic acid, the mycotoxin fumonisin or maternal exposures, such as high temperature resulting from fever (Gelineau‐van Waes et al., 2009; Wlodarczyk et al., 2012; Copp et al., 2013).

The area of most extensive study has been the potential contribution of maternal nutrition, given the observation that vitamin levels and risk of an affected pregnancy were both associated with socioeconomic status (Leck, 1974; Smithells et al., 1976; Au et al., 2010). Attention has particularly focused on vitamins relating to folate one‐carbon metabolism. Hence, maternal blood folate and vitamin B_12_ status are independent risk factors for NTDs, while elevated homocysteine, an inversely related biomarker of impaired folate status, is also associated with NTDs (Kirke et al., 1993; Blom et al., 2006).

The corollary to NTD risk conferred by sub‐optimal maternal vitamin status is that supplementation with specific nutritional factors may have a protective effect. Clinical trials have demonstrated a protective effect of periconceptional folic acid supplementation against NTD recurrence (after a first affected pregnancy) and occurrence (Smithells et al., 1980; MRC Vitamin Study Research Group, 1991; Czeizel and Dudás, 1992). The clear evidence for a preventive effect of folic acid led to recommendations that women planning pregnancy should take a folic acid supplement.

In addition, public health initiatives to implement mandatory food fortification have been associated with a reduction in NTD prevalence in many countries (Blencowe et al., 2010; Crider et al., 2011; Rosenthal et al., 2014). Nevertheless, the findings of clinical trials, monitoring of supplement use and case studies of recurrent NTDs all indicate that some NTDs are not preventable by folic acid (termed folic acid‐resistant or non‐responsive) (MRC Vitamin Study Research Group, 1991; Mosley et al., 2009; Bupp et al., 2015).

The recognition that some NTDs are not preventable by folic acid engendered interest in other nutritional factors that may contribute to prevention of NTDs. In this review, we focus on a potential role for another nutrient, inositol.

### Inositol and Its Derivatives Participate in a Diverse Range of Cellular Functions

**Figure 1:**
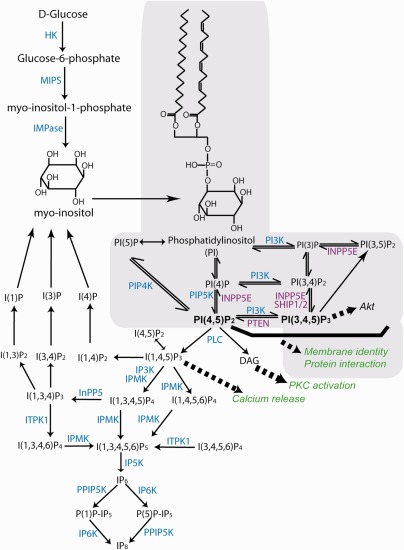
Outline of pathways for synthesis of inositol phosphates and phosphoinositides. Inositol can be obtained from dietary sources or synthesized from D‐glucose by the sequential action of hexokinase (HK), myo‐inositol‐1‐phosphate synthase (MIPS), and inositol monophosphatase (IMPas). The structure of *myo*‐inositol and phosphatidylinositol are shown (inositol lipids are shaded gray). These molecules form the backbone for synthesis of inositol phosphate (IP) and phosphoinositide (PI) molecules, mediated by action of multiple kinases and/or phosphatases. Enzymes that are discussed in the text are indicated (kinases in blue text, phosphatases in purple text). Notably, several phosphorylation/dephosphorylation steps can be mediated by several enzymes. For example, a number of kinases exist as multiple isoforms (e.g., PIP5K and PI3K [PI3‐kinase]), while several phosphatases can act to dephosphorylate PI(3,4,5)P_3_ (e.g., INPP5E, SHIP1, and SHIP2). In addition, some enzymes act at multiple steps (e.g. IPMK acts to phosphorylate inositol at a number of steps). Examples of downstream functional effects of key molecules are indicated in italics (green text). PLC, phospholipase C; DAG, diacylglycerol; PKC, protein kinase C; IPMK, inositol phosphate multikinase; ITPK1, inositol 1,3,4‐triphosphate 5/6 kinase.

Inositol (1,2,3,4,5,6‐hexahydroxycyclohexane) is a naturally occurring simple six carbon sugar alcohol, sometimes referred to as a pseudo‐vitamin (vitamin Bh or B8), although it can be synthesized from glucose by means of conversion of glucose 6‐phosphate to inositol 1‐phosphate (Fig. 1). There are nine stereoisomers of inositol, of which *myo‐*, *D‐chiro*‐, *scyllo*‐, *epi*‐, *muco*‐, and *neo*‐inositol occur naturally, with the predominant form being *myo*‐inositol (Michell, 2008; Leung et al., 2011). Adults typically consume approximately 1 g of *myo‐*inositol per day which is present in a variety of foods including nuts, vegetables, and citrus fruits (Holub, 1987; Croze and Soulage, 2013). Plants, particularly legumes, oil seeds, and grains, are particularly rich in *myo‐*inositol hexakisphosphate (IP_6_; phytic acid); this is mostly hydrolyzed to free inositol before absorption from the gut (Michell, 2008; Schlemmer et al., 2009). However, owing to its ion‐chelating properties, an excess of phytate from dietary sources could theoretically hinder absorption of cations, such as Ca^2+^, from the gut (Wilson et al., 2015). *Myo‐*inositol in the nonphosphorylated form, typically available in vitamin supplements, does not have this property. Although less abundant, D‐*chiro‐*inositol is also obtained from dietary sources, principally in the methylated form 3‐*O‐methyl‐D‐chiro*‐inositol (pinitol) (Negishi et al., 2015).

Inositol is incorporated as the polar head‐group of membrane lipids based on phosphatidylinositol (PI), which is synthesized from *myo*‐inositol and cytidine diphosphate‐diacylglycerol (CDP‐DAG). Hence, phosphorylation of inositol at carbons 3, 4, and 5 generates a series of phosphoinositides which have multiple cellular functions in signaling and membrane dynamics (Di Pietro and De Camilli, 2006). When not bound to lipids, free inositol can carry a phosphate group at each of its six carbons (IP_1_ to IP_6_). IP_1_, IP_2_, and IP_3_, are generated by hydrolysis of PIP_2_ and subsequent recycling of inositol, while more highly phosphorylated forms (IP_4_, IP_5_, and IP_6_) and pyro‐phosphates (e.g., PP‐IP_5_ equivalent to IP_7_) are generated by the action of specific kinases (Livermore et al., 2016). Hence, intracellular inositol is present in a complex variety of lipid‐bound inositides as well as soluble inositol phosphates and pyrophosphates (Fig. 1).

#### Functions of phosphoinositides and inositol polyphosphates

The phosphoinositides PI(4,5)P_2_ and PI(3,4,5)P_3_ are key mediators of major signaling pathways that influence diverse cellular functions (Fig. 1). These pathways are reviewed extensively elsewhere (Di Paolo and De Camilli, 2006; Michell, 2008; Bridges and Saltiel, 2015). In brief, hydrolysis of PI(4,5)P_2_ by phospholipase C generates the second messengers inositol tri‐phosphate (IP_3_) and diacylglycerol (DAG). IP_3_ stimulates release of intracellular calcium while DAG stimulates several isoforms of protein kinase C (Nishizuka, 1992; Berridge, 1993;). Alternatively, phosphorylation of PIP_2_ at the 3‐position of inositol by PI3‐kinases generates PI(3,4,5)P_3_ which recruits downstream targets, including the serine‐threonine kinase Akt (Burgering and Coffer, 1995; Larue and Bellacosa, 2005; Hawkins and Stephens, 2015). The regulation of PI(3,4,5)P_3_ abundance by activation of PI3‐kinases and phosphatases such as PTEN regulates a range of downstream cellular properties including proliferation, motility and polarity (Tsujita and Itoh, 2015).

In parallel with their role in signaling pathways, phosphoinositides interact with numerous proteins and thereby provide an interface between membranes and the cytosol in a wide range of cellular contexts (Janmey and Lindberg, 2004; Posor et al., 2015). Regulation of the abundance of phosphorylated forms of PI involves the concerted action of a series of different kinases and phosphatases. For example, PI(4,5)P_2_ can be generated from PI(4)P by the action of PI4P 5‐kinases or to a much lesser extent from PI(5)P by the action of PI5P 4‐kinases (Fig. 1). There are several family members for each kinase, together with several corresponding phosphatases. Hence, there appears to be a complex regulation of the abundance of differing PIs.

Localized activity of specific kinases and phosphatases is thought to generate dynamic spatially restricted enrichment of particular PIs which can modulate membrane dynamics (Janmey and Lindberg, 2004; Saarikangas et al., 2010; Cauvin and Echard, 2015). For example, coordination of endocytosis and vesicle trafficking depends on specific PI pools. Clathrin‐mediated endocytosis requires PI(4,5)P_2_, with switching between PI types providing compartment identity as vesicles progress along the endocytic route (Croise et al., 2014; Posor et al., 2015). PIs, particularly PI(4,5)P_2_, can be coupled to the underlying F‐actin cytoskeleton by means of “linker” proteins such as class I myosins and Ezrin/Radixin/Moesin proteins, while PIP_2_ can also suppress proteins such as cofilin that mediate actin disassembly (Janmey and Lindberg, 2004; Tsujita and Itoh, 2015). Modulation of PI metabolism, for example through hydrolysis of PIP_2_, can thereby contribute to local alterations in membrane‐cytoskeleton tension during cell shape changes and rearrangement (Tsujita and Itoh, 2015). These properties of various PIs are implicated in membrane ruffling and micropinocytosis (Araki et al., 2007; Posor et al., 2015) as well as several events during cell division including spindle orientation, cell rounding and abcission (Field et al., 2005; Cauvin and Echard, 2015).

Among the proteins that interact with phosphoinositides and/or link PIs to F‐actin, a number are implicated in neural tube closure and/or NTDs. For example, several of the regulatory roles of PIs involve a close functional interaction with small GTPases, including Rac1, with which they associate at the membrane. Notably, disruption of cytoskeletal structure or turnover and impaired function of small GTPases are implicated in NTDs in several mouse models (Copp and Greene, 2010; Escuin et al., 2015; Rolo et al., 2016). Loss of function of another protein, MARCKS, which links PIP_2_ to the actin cytoskeleton also results in NTDs in mice (Stumpo et al., 1995; McLaughlin et al., 2002).

### Inositol Is Required for Neural Tube Closure

#### Inositol deficiency causes cranial NTDs in mice

Interest in the influence of inositol levels on NTD predisposition was stimulated by nutritional studies in which rat embryos were cultured in defined conditions through the period of neural tube closure (Cockroft, 1979, 1988). Specific omission of several vitamins caused growth retardation or developmental abnormalities but NTDs occurred only in the absence of inositol. Similarly, deficiency of inositol in the culture medium causes cranial NTDs in mouse embryos, whereas deficiency of other vitamins, including folic acid, does not (Cockroft et al., 1992). Although inositol deficiency caused cranial NTDs among non‐mutant strains of mice, increased sensitivity was observed among cultured embryos of the *curly tail* (*ct*) strain, which are genetically predisposed to spinal and cranial NTDs (Cockroft et al., 1992). Under normal conditions *curly tail* embryos show only around 3 to 7% incidence of cranial NTDs (exencephaly). However, inositol‐deficiency caused much higher rates of cranial NTDs (∼70%) and an increased concentration of inositol was required to correct these defects than in other strains tested (Cockroft et al., 1992).

#### Inositol metabolism and neural tube defects in mouse models

Analysis of mice carrying gene‐trap alleles that result in loss or diminished expression of inositol kinases suggests that both phosphoinositides and inositol polyphosphates play key cellular functions essential for neural tube closure.

Cranial NTDs occur in embryos lacking PIP5KIγ, one of three isoforms of phosphatidylinositol‐4‐phosphate 5‐kinase that mediates synthesis of PI(4,5)P_2_ (Wang et al., 2007). Of interest, cranial NTDs also occur in mice lacking inositol polyphosphate 5‐phosphatase E (encoded by *Inpp5E*), which hydrolyses PI(4,5)P_2_ or PI(3,4,5)P_3_, and hence opposes the effect of PIP5KIγ on levels of PI(4)P (Jacoby et al., 2009). Given the functions of PI(4,5)P_2_ not only as substrate for phospholipase C‐mediated signaling by means of diacylglycerol and IP_3_ but also in membrane identity/trafficking, cytoskeletal organization and cell division, impaired production could potentially disturb neurulation through several different cellular mechanisms.

Recent studies suggest an additional possible mechanism relating to a requirement for regulation of phosphoinositides in regulating biogenesis and stability of primary cilia. PIP5KIγ localises to the basal body of cilia and appears to be essential for ciliogenesis, probably by depleting PI(4)P (Chavez et al., 2015; Xu et al., 2016). In contrast, Inpp5E opposes the action of PIP(5)KIγ during initial events of ciliogenesis (Xu et al., 2016), but appears to be required within established cilia to maintain their structural stability (Bielas et al., 2009; Plotnikova et al., 2015). Hence, phosphoinositide homeostasis appears to play a key role in cilia formation and function. In addition to cranial NTDs, mice lacking *Inpp5E* exhibit features of ciliopathies such as polycystic kidneys and polydactyly (Jacoby et al., 2009), while mutations in *INPP5E* are found in the ciliopathies Joubert Syndrome and MORM Syndrome (Bielas et al., 2009; Jacoby et al., 2009). Notably, cranial NTDs are observed in several other mouse mutants in association with abnormal cilia (Murdoch and Copp, 2010).

In addition to phosphoinositides, several functions have been ascribed to inositol phosphates beyond the function of IP_3_ in intracellular calcium signaling. In particular, generation of higher inositol polyphosphates appears to be required during the stage of neural tube closure. Mice that are deficient for *Ipmk*, encoding an inositol phosphate multikinase (Impk, also known as Ipk2), fail to develop beyond E9.5 (Frederick et al., 2005). Furthermore, embryos with reduced expression of *Itpk1*, encoding inositol 1,3,4,‐triphosphate 5/6‐kinase show growth retardation and cranial NTDs (frequency of ∼12%) (Wilson et al., 2009). These inositol kinases are crucial for production of the highly phosphorylated forms of inositol IP_4_, IP_5_, and IP_6_, the most abundant of which within cells is inositol hexakisphosphate (IP_6_) (Wilson et al., 2015). Impk may also act as a PI3‐kinase and can mediate transcriptional regulation through kinase‐independent functions (Kim et al., 2015).

Intriguingly, IP_4_ has been found to play a key role in modulating Rho GTPase activity and F‐actin during epithelial repair in *Xenopus* embryos (Soto et al., 2013). Given the requirement for Rho kinase‐dependent regulation of actomyosin turnover during mammalian neurulation (Escuin et al., 2015), it is tempting to speculate that inositol polyphosphates could directly influence the closure process.

#### Glycosylphosphatidylinositol‐anchored proteins and NTDs

In addition to PIs and IPs, inositol forms a structural component of glycophosphoinositide (GPI) anchors that link many different proteins to cell surfaces (Englund, 1993; Paulick and Bertozzi, 2008). Inositol glycans, derived from hydrolysis of GPI‐anchors, are also proposed to act as second messengers in certain signaling reactions responsive to insulin (Nascimento et al., 2006; Croze and Soulage, 2013).

Although GPI‐anchors may represent only a small proportion of cellular inositol it is intriguing that cranial NTDs arise in knockout mice for genes encoding several different GPI‐anchored proteins including *Rgma* (repulsive guidance molecule A; Niederkofler et al., 2004), *Efna5* (ephrinA5) (Holmberg et al., 2000), and *Folr1* (folate receptor 1, folate‐binding protein1; Piedrahita et al., 1999). Moreover, treatment of cultured embryos with phosphatidylinositol‐specific phospholipase C, which cleaves GPI anchors, disturbs spinal neural tube closure (O'Shea and Kaufman, 1980; Abdul‐Aziz et al., 2009).

### Inositol Supplementation in Models of NTDs

Uptake of inositol by the embryo and generation of inositol phosphoinositides and inositol polyphosphates are required for cranial neurulation (Table 1). This raises the question of whether supplemental inositol may prevent NTDs in some models.

**Table 1:** Experimental Models in Which Inositol Status or Metabolism Is Associated with Neural Tube Closure

Inositol‐related deficits in NTD causation		NTD type	Comments/mechanism	Reference
Inositol deficiency	Cultured mouse or rat embryos	Exencephaly	NTDs in non‐mutant and mutant strains. Higher incidence in *ct* strain	Cockroft, 1992
PI4P5KIγ null	PIP kinase generates PI(4,5)P_2_	Exencephaly	Disordered actin Cilia defect?	Wang, 2007
Inpp5e null	PI(4,5)P_2_ and PI(3,4,5)P_2_ phosphatase	Exencephaly	Unstable cilia and ciliopathy phenotypes	Jacoby, 2009
Itpk1 hypomorph	Generates IP(1,3,4,5)P_4_ and IP(1,3,4,6)P_4_	Exencephaly &/or spina bifida	Deficit of higher IPs	Wilson, 2009
NTD prevention by inositol in experimental models				
*Curly tail* mouse	Embryo culture; Oral; I.P. injection; sub‐cutaneous route	Spina bifida	Corrects proliferation defect. Requires activity of PKC isoforms. *Chiro‐*inositol more effective than *myo‐*inositol	Greene, 1997; Cogram, 2002; Cogram, 2004
Hyperglycemia	High glucose in embryo culture	Exencephaly	Restores inositol levels. Arachidonic acid signalling?	Baker, 1990; Hashimoto, 1990;
Diabetes	Streptozotocin‐induced diabetes	Exencephaly	Restores inositol levels?	Khandelwal, 1998
Folate‐deficient NTDs	Dietary folate deficiency in wild‐type strain (*ct* genetic background)	Exencephaly	Unknown	Burren, 2010

#### Prevention of spina bifida in the curly tail mouse

Although inositol deficiency was found to exacerbate cranial NTDs (see above), the main neural tube closure phenotype in the *curly tail* strain involves a defect of spinal neurulation affecting approximately 50% of embryos. Failure to complete closure of the posterior neuropore (PNP) at the end of spinal neurulation results in spina bifida in around 15% of embryos. A further 35 to 40% exhibit only a tail flexion defect, a phenotype which results from delayed completion of PNP closure (Van Straaten and Copp, 2001). Unlike in the cranial region, inositol deficiency did not further exacerbate the progression of spinal neurulation in cultured *curly tail* embryos or delay closure in the nonmutant CBA/Ca strain (Greene and Copp, 1997). However, increasing the *myo‐*inositol concentration normalized PNP closure in cultured embryos (Greene and Copp, 1997). This protective effect was confirmed by in vivo supplementation by means of maternal intra‐peritoneal injection, oral dosing or subcutaneous infusion (Greene and Copp, 1997; Cogram et al., 2002), with each strategy leading to a significant reduction in the frequency of spina bifida.

Interestingly, an alternative enantiomer of inositol, *D‐chiro‐*inositol showed a greater protective effect than *myo‐*inositol, with a larger reduction in incidence of spina bifida at the same dose, and maintained effectiveness even at a lower dose (Cogram et al., 2002). Whether *myo‐* and *chiro*‐inositol act through the same mechanism is still unknown. Interconversion of the two enantiomers can be mediated by an endogenous epimerase (Pak et al., 1992; Sun et al., 2002), although other groups report limited interconversion in vivo (Lin et al., 2009). Notably, NTDs in the *curly tail* model are not preventable by folic acid and provide a model for “folic acid‐resistant” NTDs. Identification of a protective therapy such as inositol, therefore, raises the question of whether this approach may also have utility in preventing some human NTDs.

Supplemental inositol could potentially act to overcome the underlying causative defect responsible for NTDs and/or enhance the normal processes required for progression of neural tube closure. Completion of PNP closure does not occur prematurely in nonmutant embryos supplemented with inositol (Greene and Copp, 1997), which would argue that inositol may prevent NTDs by normalizing an underlying developmental abnormality. In the *curly tail* model, spina bifida results from a proliferation defect in the hindgut endoderm (Copp et al., 1988). This creates a growth imbalance between dorsal and ventral tissues, leading to increased ventral curvature of the caudal region of the embryo that mechanically opposes closure leading to spina bifida (Brook et al., 1991). This defect is thought to result from reduced expression of the transcription factor *Grhl3*. The *curly tail* strain is homozygous for a hypomorphic allele of *Grhl3* and reinstatement of *Grhl3* expression normalizes spinal neurulation (Ting et al., 2003; Gustavsson et al., 2007).

However, penetrance of defects is also strongly influenced by modifiers in the genetic background, such as a polymorphic variant of lamin B1 (de Castro et al., 2012). Inositol supplementation increases proliferation in the hindgut endoderm of *curly tail* embryos, which would explain the preventive effect on spinal NTDs (Cogram et al., 2004). Consistent with this hypothesis, spina bifida was also found to be preventable by combinations of thymidine and purine nucleotides, which also normalize proliferation in the hindgut (Leung et al., 2013).

In cultured embryos labelled inositol is taken up and incorporated into inositol phosphates and inositol‐containing phospholipids. This appears to be necessary for inositol's protective effect in *curly tail* embryos as inhibition of inositol phosphate recycling abolishes the protective effect (Greene and Copp, 1997). Increased production of PtdIns(4,5)P_2_ could potentially have a direct ameliorating effect on the underlying proliferation defect in *curly tail* embryos, owing to the its functions in cell division (Cauvin and Echard, 2015). In addition, among potential downstream effects following PtdIns(4,5)P_2_ hydrolysis, activation of protein kinase C appears to play a key role in prevention of spinal NTDs because the protective effect of inositol is (i) mimicked by short‐term treatment with a phorbol ester activator of PKC, and (ii) abrogated by broad spectrum and isoform‐specific PKC inhibitors (Greene and Copp, 1997; Cogram et al., 2004).

#### Inositol does not prevent NTDs in all models

The protective effect of inositol against spina bifida is not universal in mouse NTD models. For example, while prevention of NTDs in the *curly tail* model was confirmed in an independent laboratory, no response was observed among *Grhl3* null mice (Ting et al., 2003). This is likely due to the greater severity of NTDs, which are fully penetrant in the complete knockout of *Grhl3* compared with diminished expression in the *curly tail* hypomorph. In the context of cranial NTDs, it is also possible that inositol acts in the *curly tail* strain to normalize effects of genetic modifiers rather than the effects of *Grhl3* mutation. For example, cranial NTDs in the *curly tail* strain appear to largely be a feature of the genetic background as, unlike spina bifida, they are not prevented by transgenic reinstatement of *Grhl3* expression (Sudiwala et al., 2016). Spinal closure defects in the *Bent tail* mouse, carrying an X chromosome deletion that encompasses *Zic3* (Franke et al., 2003; Klootwijk et al., 2004), and in *Mekk4* knockouts (Chi et al., 2005) were also found to be unresponsive to inositol treatment.

#### Inositol and susceptibility to NTDs conferred by maternal diabetes

NTDs occur in cultured rat and mouse embryos exposed to hyperglycemia or streptozotocin‐induced diabetes (Sadler, 1980; Cockroft, 1984), both of which elicit depletion of tissue inositol levels (Hod et al., 1986; Sussman and Matschinsky, 1988; Hashimoto et al., 1990). Supplementation with inositol in cultured embryos corrects the hyperglycemia‐induced growth retardation and NTDs (Baker et al., 1990; Hashimoto et al., 1990; Hod et al., 1990). Similarly, oral administration of *myo*‐inositol led to a reduction in the frequency of diabetes‐induced abnormalities in rat embryos (Khandelwal et al., 1998) (Table 1).

Supplemental inositol restores tissue inositol levels, but could plausibly also act through pathways downstream of inositol phosphates or phosphoinositides. Arachidonic acid can be derived from diacylglycerol which is produced upon hydrolysis of PIP_2_. The protective effect of inositol against hyperglycemia in vitro is reversed by indomethacin, an inhibitor of arachidonic acid metabolism (Baker et al., 1990). Conversely, arachidonic acid and some derivative prostaglandins have a protective effect against NTDs in the hyperglycinemia and streptozotocin models (Goldman et al., 1985; Pinter et al., 1986). Whether inositol acts to prevent hyperglycemia and/or diabetes‐related NTDs through stimulating arachidonic acid signaling, upstream of prostaglandins and leukotrienes, or this pathway is required in parallel with inositol for cranial neurulation is not clear. Unlike, phorbol esters, arachidonic acid did not substitute for inositol in prevention of spinal NTDs in the *curly tail* model (Greene and Copp, 1997).

#### Prevention of NTDs resulting from folate deficiency?

While inositol reduces NTD frequency in the *curly tail* mouse, in which folic acid is not preventive, it is important to ask whether inositol can also prevent NTDs in models that are responsive to folic acid. Supplemental folic acid has been found to reduce the frequency of NTDs resulting from mutation of the *Pax3* transcription factor in the *Sp^2H^* and *Sp* (alleles of *Splotch* mice) models (Fleming and Copp, 1998; Wlodarczyk et al., 2006), whereas *myo*‐inositol was not protective (Wlodarczyk et al., 2006). In dietary models, folate‐deficiency does not cause NTDs unless in the presence of a predisposing genetic mutation or background, such as *Pax3* deficiency (Burren et al., 2008; Heid et al., 1992). Embryos with a genetic background similar to the *curly tail* strain but wild‐type for *Grhl3* (+*^ct^/*+*^ct^*) do not develop cranial NTDs under normal dietary conditions but exhibit exencephaly when made folate‐deficient (Burren et al., 2010). As expected, NTDs in this model can be prevented by maternal administration of folic acid. Notably, however, *myo‐*inositol has a similar protective effect (Burren et al., 2010), raising the possibility that in some circumstances inositol could overcome NTD‐predisposing abnormalities imposed by sub‐optimal folate status. In this context, we cannot currently rule out a role for inositol, by means of its GPI‐anchor function, in promoting enhanced folate uptake by means of Folr1.

### Inositol Status and Human NTDs

Few studies have systematically determined maternal inositol status in relation to NTD risk. Estimates of dietary *myo*‐inositol intake from a food frequency questionnaire did not suggest a strong association with risk of NTDs (Shaw et al., 2005). Moreover, determination of serum *myo‐*inositol concentration in mothers and their infants with spina bifida did not show significant variation from controls (63–102 subjects in each group), although a trend toward lower values was recognized (Groenen et al., 2003). However, among the lowest maternal serum *myo*‐inositol concentrations (lowest decile), there was a significant association with having a child with spina bifida (Groenen et al., 2003). If these findings are representative of maternal inositol status during pregnancy, one could speculate that insufficient inositol may contribute to development of NTDs in the offspring. Further studies on this relationship are warranted as evidence suggests that blood *myo*‐inositol levels may change during pregnancy (Groenen et al., 2006). Of note, in a more recent study in which samples were collected during pregnancy, maternal plasma IP_6_ was found be significantly diminished among NTD cases compared with controls (60 in each group) (Guan et al., 2014).

Considering potential genetic influences on inositol uptake or metabolism, analysis of variants in a *myo*‐inositol transporter, *SLC5A11*, did not suggest an association with spina bifida in a Dutch population (Groenen et al., 2004). However, investigation of maternal *ITPK1* genotype, whose deficiency can cause NTDs in mice (Wilson et al., 2009), suggests a potential role in NTDs. A single nucleotide polymorphism (SNP) ‐based case–control analysis in a Chinese population, revealed a significant association of NTD with four tag SNPs in maternal *ITPK1* that was principally attributable to association with spina bifida. Among the associated SNPs, rs3783903 also appeared to be predictive of lower *ITPK1* expression in blood and plasma inositol levels (Guan et al., 2014).

### Inositol Supplementation in Human Pregnancy

The findings of a preventive effect of a nutrient supplement, such as inositol, in rodent NTD models raises several questions, including: (1) are inositol supplements safe to use during pregnancy in humans, and (2) could inositol prevent some human NTDs?

In mouse studies, neither *myo*‐ nor *chiro*‐inositol had notable side‐effects in the supplemented dams or in the developing fetuses (Cogram et al., 2002). As described above, inositol supplementation has beneficial effects in several NTD models without apparent deleterious effects. However, in mice treated with valproic acid, an anti‐epileptic with known teratogenic properties, inositol was found to exacerbate the induced developmental abnormalities (Massa et al., 2006). Use of anti‐epileptics was an exclusion criterion in a subsequent clinical trial of inositol during pregnancy (Greene et al., 2016).

In adult humans, inositol has been tested for a variety of conditions (Table 2), including psoriasis, panic disorder, depression, and eating disorders (Benjamin et al., 1995; Levine et al., 1995; Chengappa et al., 2000; Gelber et al., 2001; Allan et al., 2004). Daily dosage ranging from 6 g to as high as 18 g did not result in major side effects, although mild flatulence or diarrhea was reported by a few patients. No side effects have been reported for doses less than 6 g per day. Overall, the outcomes of studies in adults do not suggest that supplemental inositol has deleterious effects (Croze and Soulage, 2013).

**Table 2:** Clinical Use of *myo‐*Inositol

Condition	Study size (total inositol and placebo groups)	Dose (daily, *myo*‐inositol unless stated)	Outcome	References
Adult conditions				
Psoriasis in patients taking lithium	15	6 g for 1 week	Beneficial effect in psoriasis	Allan, 2004
Bulimia nervosa and binge eating	12	18 g for 6 weeks	Indication of therapeutic value	Gelber, 2001
Add‐on treatment for bipolar disorder	24	12 g 6 weeks	Indication of beneficial effect	Chengappa, 2000
Panic disorder	21	12 g for 4 weeks	Decline in frequency & severity	Benjamin, 1995
Depression	28	12 g for 4 weeks	Improved score in depression scale	Levine, 1995
Metabolic syndrome	80	2 g for 6 months	Improved blood pressure parameters	Giordano, 2011
Treatment prior to or during pregnancy				
Polycystic ovary syndrome	20	2 g for 12 weeks	Improved insulin sensitivity & menstrual cycle activity	Genazzani, 2008
Polycystic ovary syndrome	92	4 g for 14 weeks	Impoved ovarian function	Gerli, 2007
Polycystic ovary syndrome	42	4 g for 12–16 weeks	Improved insulin sensitivity & ovulation	Costantino, 2009
Elevated fasting glucose	75	4 g throughout pregnancy	Reduced incidence of gestational diabetes	Matarrelli, 2013
Risk of gestational diabetes	220	2 g from 1^st^ trimester	Reduced incidence of gestational diabetes	D'Anna, 2013
Gestational diabetes	69	4 g for 8 weeks	Decline in fasting glucose	Corrado, 2011
NTDs ‐ recurrence	12 non‐randomised (15 pregnancies)	0.5 or 1 g daily to 60 days pregnancy	No recurrent NTDs (0/17 babies)	Cavalli and Copp, 2002 Cavalli, 2011
NTDs ‐ recurrence	47 randomised 22 non‐randomised	1 g daily to 12 weeks pregnancy (+ folic acid)	No NTDs among pregnancies in inositol groups (14 randomised and 21 non‐randomised). 3 NTDs in non‐supplemented groups	Greene, 2016

Examples of conditions in which *myo*‐inositol has been tested for potential beneficial effects.

An important additional consideration in any potential novel periconceptional preventive therapy for NTDs is the requirement for safety in the fetus and pregnant mother. The inositol concentration is higher in reproductive organs than in blood, and concentration in the ovaries is under hormonal control (Lewin et al., 1982). Several clinical trials in women with polycystic ovary syndrome have suggested a beneficial effect of inositol (*myo‐* or *chiro‐*) supplements in improving ovarian function, hormone status and menstrual activity, with no reported side effects at typical doses of 2 to 4 g daily (Table 2; Nestler et al., 1999; Gerli et al., 2007; Genazzani et al., 2008; Costantino et al., 2009; reviewed by Dinicola et al., 2014).

Furthermore, similar doses of inositol have been trialed in randomized studies during pregnancy for treatment and/or prevention of gestational diabetes (Corrado et al., 2011; D'Anna et al., 2013; Matarrelli et al., 2013), as well as in postmenopausal women with metabolic syndrome (Giordano et al., 2011). These studies were based on the hypothesis that inositol would be beneficial, as *myo‐* and D‐*chiro‐*inositol and their methylated derivatives (sequoyital and D‐pinitol, respectively) were reported to have insulin‐sensitizing properties in experimental models of hyperglycemia and diabetes (Croze and Soulage, 2013). In women considered at risk owing to a family history of type 2 diabetes (D'Anna et al., 2013) or elevated fasting glucose (Matarrelli et al., 2013), there was a lower incidence of gestational diabetes in the *myo*‐inositol group compared with placebo. Supplementation (2 g inositol, twice daily) throughout pregnancy from the first trimester onward was not associated with side effects or increased risk of adverse pregnancy outcomes (reviewed by Croze and Soulage, 2013; Noventa et al., 2016).

### Prevention of NTDs by Inositol?

Taking into account the prevalence of NTDs, the sample size required for investigation of a potential novel therapy in a “first occurrence” trial is likely to be prohibitively large. As a result, inositol supplementation has so far been studied solely in women with one or more previous NTD‐affected pregnancies (Table 2). Such women are known to be at considerably higher risk of a subsequent NTD‐affected pregnancy than women without a history of NTDs: the recurrence risk after a single affected pregnancy is typically quoted as 3.1%, rising to 11.8% after two affected pregnancies (Seller, 1981). A cohort of 12 women in Italy took *myo*‐inositol and folic acid pre‐ and postconceptionally after at least one previous affected pregnancy. Folic acid use was reported in the majority of their previous pregnancies which suggests that most of these NTDs were “folic acid‐resistant”. Fifteen subsequent pregnancies were followed up and 17 babies were born without NTDs (Cavalli and Copp, 2002; Cavalli et al., 2011). These findings encourage the view that there may be a contribution of inositol toward prevention of NTD recurrence. However, the number of pregnancies was too small to draw firm conclusions and the nonrandomized study design was a potential source of bias.

Where ethically and logistically possible, a randomized controlled trial is generally considered the optimal approach to determine the efficacy of a novel therapy. Given the known protective effect of folic acid, it would be unethical to conduct a trial without providing it to all participants. Hence, a potential trial design would test whether inositol + folic acid is more effective than placebo + folic acid. The MRC Vitamin Study recruited around 1800 women who had a previous NTD‐affected pregnancy and the outcome of 1200 subsequent pregnancies was ascertained (approximately 600 in the combined folic acid supplemented groups and 600 in combined nonfolic acid groups). The recurrence rate in the folic acid groups was 1% compared with 3.5% in the other groups. Detection of a further significant reduction in recurrence rate, due to inositol supplementation, should require a similar or larger sample size.

Another consideration is that any trial for prevention of NTDs now takes place in the context of use of folic acid supplement use and/or food fortification on a population level, including among women without a previous history of NTDs. Hence, it could be hypothesized that those NTDs which occur now may be more enriched for folic acid–non‐responsive NTDs than at the time of the folic acid trials in the 1980s, when use of folic acid supplements was not widespread. Similar lack of response in a subsequent pregnancy would thus lead to a higher rate of recurrence than in the MRC Vitamin Study. However, in the United Kingdom, it is estimated that only around 30 to 35% of women take periconceptional folic acid (Bestwick et al., 2014) and food fortification is not in place. Therefore, the likely recurrence rate in a folic acid supplemented study group is difficult to predict.

A pilot double‐blind randomized clinical trial of inositol was performed in the United Kingdom, with recruitment during 2009 to 2013 to make further progress toward determining whether use of inositol supplements with folic acid would have a greater protective effect than folic acid alone. The PONTI (Prevention Of Neural Tube defects by Inositol) study recruited women with one or more previous affected NTD pregnancies, who were planning a further pregnancy (Greene et al., 2016). The study design involved randomization of equal numbers of pregnancies to inositol (1 g daily) + folic acid (5 mg daily) or placebo + folic acid groups and, among 99 eligible women who contacted the study center, 47 agreed to be randomized. The majority of randomized women (46 of 47) had a history of one previous affected pregnancy (31 spina bifida and 15 anencephaly; one woman had two previous pregnancies with anencephaly). Among 14 established pregnancies in the inositol + folic acid group, all led to the birth of unaffected babies. Among 19 established pregnancies in the placebo + folic acid group, 18 led to normal outcomes, while one fetus was diagnosed with anencephaly on ultrasound (Greene et al., 2016).

One aim of the study was to evaluate the feasibility of recruiting women at “high risk” of an NTD pregnancy into a randomized study. In fact, many women (around half of those who contacted the study team) were unwilling to enter the randomized study; almost all cited the possibility of inclusion in the placebo group as their reason for declining randomization. This was despite provision of folic acid to all participants and the unproven nature of inositol's preventive effect. A cohort of 22 women who met the eligibility criteria for the PONTI study but decided against randomization, agreed to be followed up in subsequent pregnancies. Among this nonrandomized group 19 women, 6 of whom had experienced two previous affected pregnancies, used peri‐conceptional supplementation with folic acid (at least 5 mg daily) and inositol (typically 1 g daily). They reported 21 pregnancies without NTDs. A further three nonrandomized women, who subsequently took only 5 mg folic acid reported one normal pregnancy outcome and two pregnancies that were terminated following a diagnosis of anencephaly.

### Conclusions and Future Prospects

Inositol, inositol phosphoinsitides, and inositol polyphosphates are a group of molecules that fulfil a diverse range of cellular functions. Supply and metabolism of inositol is required for cranial neural tube closure, while supplemental inositol can prevent spinal and cranial NTDs in various experimental models. Notably, these include the *curly tail* strain, whose multigenic causation perhaps models the etiology of human NTDs more representatively than single gene knockouts. In addition, inositol has been implicated in ameliorating NTDs in diabetes‐induced and folate‐deficiency models, each of which are implicated in human NTDs. Exogenous inositol could potentially act to overcome the underlying causative defect responsible for NTDs and/or enhance the normal processes required for progression of neural tube closure. Indeed, in the curly tail model, inositol has been found to counteract the genetically determined imbalance of cell proliferation in the region of caudal neurulation. Given the multiple functions of inositol in differing cellular signaling pathways and processes it is also plausible that there are differing metabolic and cellular mechanisms underlying the effects of inositol in the cranial and spinal regions and in the various models where it has been shown to act. Further studies will address these questions.

Clinical use of inositol in adults and during pregnancy does not indicate safety concerns at the doses tested to date and beneficial effects on development of gestational diabetes have been reported. In the context of NTD prevention, the findings of recent non‐randomized and randomized studies are encouraging but these studies are currently of insufficient size to demonstrate a significant protective effect of inositol. Moreover, studies to date have been recurrence trials so the effect of inositol on first occurrence of NTDs has not yet been formally investigated. We propose that a fully powered randomized controlled study is justified to test whether inositol + folic acid has a greater protective effect against NTDs than folic acid alone. Such a study will likely require a multi‐site, multi‐country design. Nevertheless, if inositol does prove to be protective it is a simple, inexpensive compound that could potentially be used on a population‐wide level, to further enhance primary prevention of NTDs.
